# Replacement of the Y450 (c234) phenyl ring in the carboxyl-terminal region of coagulation factor IX causes pleiotropic effects on secretion and enzyme activity

**DOI:** 10.1016/j.febslet.2013.08.019

**Published:** 2013-10-01

**Authors:** Alessio Branchini, Matteo Campioni, Maria Gabriella Mazzucconi, Francesca Biondo, Rosella Mari, Maria Patrizia Bicocchi, Francesco Bernardi, Mirko Pinotti

**Affiliations:** a Department of Life Sciences and Biotechnology and LTTA Center, University of Ferrara, Ferrara, Italy; b University of Rome “La Sapienza”, Umberto I Hospital, Roma, Italy; c Department of Cellular Biotechnology and Haematology, University La Sapienza, Roma, Italy; d Haemostasis & Thrombosis Center, University of Ferrara, Ferrara, Italy; e Haemophilia & Thrombosis Centre, G. Gaslini Institute, Genova, Italy

**Keywords:** Missense mutations, Carboxyl-terminal region, Impaired secretion, Dysfunctional enzyme, Gene expression, Coagulation factor IX

## Abstract

The interplay between impaired protein biosynthesis and/or function caused by missense mutations, particularly in relation to specific protein regions, has been poorly investigated. As model we chose the severe p.Y450C mutation in the carboxyl-terminal region of coagulation factor IX (FIX) and, by expression of a panel of recombinant variants, demonstrated the key role of the tyrosine phenyl group for both FIX secretion and coagulant activity. Comparison among highly homologous coagulation serine proteases indicate that additive or compensatory pleiotropic effects on secretion and function by carboxyl-terminal mutations produce life-threatening or mild phenotypes in the presence of similarly reduced protein amounts.

## Highlights

• Disease-causing missense mutations mainly impair protein biosynthesis and/or function.
• The p.Y450C mutation in factor IX (FIX) provided a model to study their interplay.
• The mutation in the carboxyl-terminus impairs both FIX protein secretion and activity.
• The phenyl group at this relatively conserved position (c234) has a key role.
• The differential effects have pathophysiological and evolutionary implications.

## 1 Introduction

The main mechanisms through which amino acid changes produce human disease forms consist of quantitative reduction of the protein amount and/or synthesis of dysfunctional molecules. The combination of these effects accounts for the overall extent of the reduction of functional protein levels, which has major pathophysiological relevance. However, due to the limited availability of informative model mutations, the interplay between these pathogenic molecular mechanisms has been poorly elucidated, particularly in relation to specific protein regions.

The features of coagulation factor IX (FIX) deficiency (Hemophilia B) make this disorder an ideal model to address this issue. The extensively characterized mutational pattern (http://www.factorix.org/)[1], [2], [3] of *F9* gene [4] displays a large predominance of missense mutations (>65%), mainly occurring in the chymotrypsin-like catalytic domain. Notwithstanding, only few pathogenic molecular mechanisms have been detailed [5], [6], [7], [8]. Strikingly, these changes are responsible for the most variable clinical phenotypes, ranging from asymptomatic to life-threatening conditions. It is worth noting that Hemophilia B, having an X-linked inheritance pattern, strongly favors the investigation of the relationship between molecular defects in the hemizygous condition and the underlying residual function. The latter is of extreme importance in bleeding disorders since even low amounts of functional protein in plasma substantially ameliorate the bleeding phenotype in patients [9]. Moreover, being FIX a secreted serine protease, its protein concentration and enzymatic activity can be finely measured by biochemical assays.

**Fig. 1:**
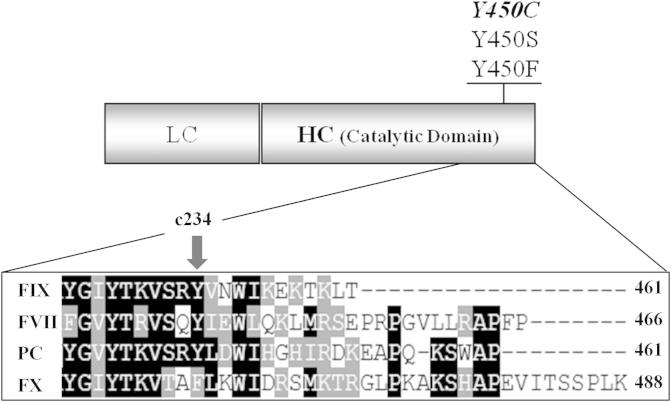
Recombinant FIX variants and sequence alignment of highly homologous coagulation factors. Schematic representation of the FIX structure with the natural (in bold) and artificial amino acid substitutions reported on the top. LC, light chain; HC, heavy chain. The box reports the sequence alignment of the carboxyl-terminal region of FIX, FVII, PC and FX and the numbers indicate the protein residues including the pre-propeptide sequence. The arrow indicates the position (c234, chymotrypsin numbering) of the FIX mutation under study.

Within the family of the coagulation serine proteases, FIX is highly homologous to factor VII (FVII), factor X (FX) and protein C (PC) [10]. In spite of their similarities, these proteins display remarkable differences both in length and amino acid composition of the carboxyl-terminal region (Fig. 1), which could underlie different structural and/or functional roles. Previous studies indicated that the carboxyl-terminus has an essential role for the secretion of FIX [11] and also of FVII [12] and PC [13]. On the other hand, alterations in this region resulted in poor secretion of normal or hyperactive variants of PC and FVII [14].

Taken together, these features make missense mutations in the carboxyl-terminal region of coagulation factors of particular interest for the investigation of the potential interplay between impaired biosynthesis and enzymatic function.

In this study, we characterized the p.Y450C missense change in the carboxyl-terminus of FIX (chymotrypsin numbering c234). By expression of a panel of recombinant FIX variants and evaluation of the secreted protein and activity we (i) dissected the contribution of the p.Y450C missense change to the extent of reduction of functional protein amounts, (ii) provided evidence for the crucial role of the phenyl ring at this position in the FIX carboxyl-terminal region and (iii) indicated major functional differences among mutations in the carboxyl-terminus of highly homologous serine proteases.

## 2 Materials and methods

### 2.1 Patient

The propositus was referred to the coagulation centre at 1 year of age because of a spontaneous hematoma. Coagulation laboratory assays indicated FIX coagulant (FIXc) activity below 1% and circulating protein levels of 0.6% (30 ng/ml) of pooled normal plasma (PNP). The family history of bleeding was negative. Upon development of an initial target joint (left ankle), he entered prophylaxis with recombinant FIX concentrate (Benefix, Baxter, 50 IU/kg) once a week, which prevented bleedings and the related complications.

Sequencing of *F9* gene [4] identified the c.1349 A > G in exon 8 that predicts the Y450C amino acid substitution in the FIX serine protease domain. This mutation has been previously found in severe Hemophilia B patients with undetectable FIXc levels [15], [16].The parents gave informed consent to conduct the study, which was in accordance with the Helsinki Declaration.

### 2.2 Expression vectors and transfection

Expression vectors for the recombinant FIX (rFIX) variants were produced by site-directed mutagenesis using the QuickChange® II Site-Directed Mutagenesis Kit (Stratagene, La Jolla, CA, USA). The mutations (referred as the underlined letter in the primer sequences) were introduced into the human FIX cDNA cloned into the pCMV5 vector [8] using the following forward primers: 5′CAAGGTATCCCGGTGTGTCAACTGG3′ (Y450C), 5′AAGGTATCCCGGTCTGTCAAC3′ (Y450S), and 5′AAGGTATCCCGGTTTGTCAAC3′ (Y450F). Reverse primers were complementary to the forward oligonucleotides. Direct sequencing confirmed the presence of the desired mutations.

Expression vectors for FIX variants were transiently transfected in Human Embryonic Kidney (HEK293) cells as previously described [8]. Forty-eight hours later, conditioned media were collected and cells lysed using a non-reducing lysis buffer (25 mM Tris–phosphate, 10% glycerol, 1% Triton® X-100, pH 7.8) supplemented with a protease inhibitor cocktail (Sigma–Aldrich, St. Louis, MO, USA).

### 2.3 FIX protein and activity assays

FIX protein levels in plasma and in conditioned medium were evaluated by ELISA (Affinity Biologicals™ Inc., Canada) using serial dilutions of a PNP as reference curve. Comparison with known amounts of purified plasma-derived FIX (Haematologic Technologies Inc., Essex Junction, VT, USA) indicated that FIX protein concentration in the PNP aliquots used in all assays was 4.5 ± 0.1 μg/ml, and this mean value was used to calculate the FIX concentration.

Western blotting analysis was conducted by SDS–PAGE on 4–15% polyacrilamide precast gels (Bio-Rad, Hercules, CA, USA). A HRP-conjugated polyclonal anti-human FIX antibody (Affinity Biologicals™ Inc.) and the enhanced chemiluminescence (ECL) reagent (Pierce®, Thermo Scientific, Rockford, IL, USA) were exploited for the detection of rFIX proteins.

FIX coagulant activity in patient plasma was assessed by standard activated partial thromboplastin time (aPTT)-based coagulation assays. To measure the coagulant activity of rFIX variants, FIX-depleted plasma (HemosIL, Instrumentation Laboratory, Lexington, MA, USA) was supplemented with rFIX-containing conditioned medium followed by aPTT assays [17]. Coagulation times were measured upon addition of a contact activator (SynthASil, Hemosil) and CaCl_2_ on a ACLTOP700 instrument (Instrumentation Laboratory). Coagulation times from serial dilutions of rFIX-wt were used to create a standard curve, which was optimized for the determination of low activity levels. The specific activity of rFIX variants was calculated as the ratio between coagulant activity and protein levels expressed as % of the rFIX-wt used in the coagulation assays. To obtain the standard deviation, we assessed the activity in media from three independent transfections for each mutant, and each medium has been evaluated in duplicate.

## 3 Results and discussion

The *F9* p.Y450C missense mutation identified by us in a Hemophilia B patient was chosen as model to investigate the interplay between the pathogenic molecular mechanisms impairing protein biosynthesis and enzymatic activity caused by alterations of the variable carboxyl-terminus of coagulation serine proteases. We took advantage from the expression of recombinant FIX variants evaluated through immunological and coagulation assays, an approach that we previously exploited to dissect the molecular bases of severe coagulation factor deficiency forms also caused by potentially “null mutations” [8], [14], [18].

### 3.1 The disease-associated p.Y450C mutation reduces secretion of a dysfunctional FIX molecule

**Fig. 2:**
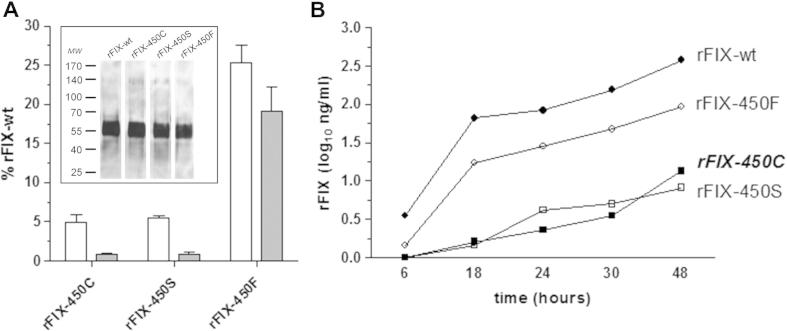
Expression levels of the recombinant FIX variants (A) Protein (white bars) and coagulant activity (grey bars) levels of secreted rFIX variants. Results are expressed as the percentage of rFIX-wt, and are reported as mean ± standard deviation from three independent experiments. Inset. Western Blotting analysis in non-reducing conditions of rFIX variants in cell lysates. MW, molecular weight marker. (B) Secreted levels of the rFIX-450C (■), rFIX-450S (□), rFIX-Y450F (○) and rFIX-wt (●) variants in time-course experiments. Conditioned media were collected at 6, 18, 24, 30 and 48 h after transfection. Protein levels are expressed as the log10 of concentration.

Transient transfection experiments indicated that the introduction of the p.Y450C mutation into the human FIX cDNA (Fig. 1) resulted in markedly reduced secreted amounts of rFIX-450C molecules, as indicated by the protein concentration in medium of 18.9 ± 6.2 ng/ml, corresponding to 4.9 ± 1.1% of those of rFIX-wt (373.8 ± 119.6 ng/ml)(Fig. 2A).

The observation that protein concentration of the secreted rFIX-450C variant was higher than that measured in patient plasma (0.6% of PNP, 30 ng/ml) might underlie a reduced stability into the circulation and/or a preferential removal of the FIX mutant molecules in vivo, which cannot be properly assessed in cellular models. On the other hand, the accumulation of the rFIX-450C in medium may have led to overestimate the secreted levels. We therefore conducted a time-course experiment. While the amount of rFIX-wt was clearly appreciable in medium even at 6 h post-transfection (3.4 ± 0.2 ng/ml), that of the rFIX-450C variant became barely detectable only at 18 h and slowly increased over time (Fig. 2B). This observation indicates a remarkable delayed secretion of the mutant FIX and, taking into account the one day half-life of FIX [19], provides a plausible explanation for the very low amount of circulating FIX in the patient.

**Fig. 3:**
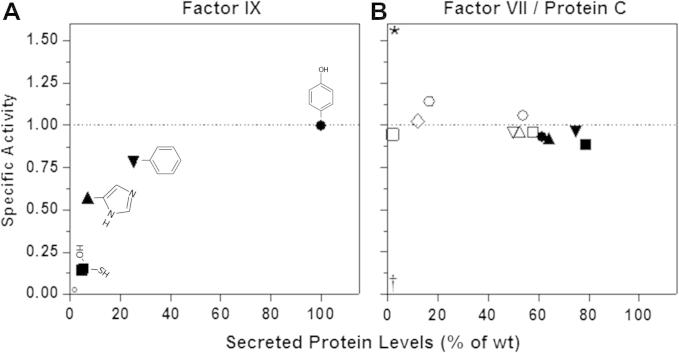
Relationship between secreted protein levels and specific activity of FIX, FVII and PC carboxyl-terminal variants. The specific activity was calculated as the ratio between activity and protein levels. The wild-type specific activity (referred as 1.00) is indicated by the dotted line. (A) FIX variants are: rFIX-wt (●), rFIX-450C (■), rFIX-450S (□), rFIX-450F (▾) and rFIX-450H variant (▴) [11]; °, rFIX-450P variant (not detectable, [11]). The rFIX-450C and rFIX-450S are superposed. The chemical structure of the side chains at position 450 is displayed aside. (B) FVII variants are: rFVII-466X (□), rFIX-465X (○) rFVII-464X (♢),rFVII-463X () rFVII-462A (○), rFVII-462 W (△), rFVII-462Q (∇). ^∗^, gain-of-function R462X variant [14]. PC variants [13] are: rPC-459X (■), rPC-456X (▾), rPC-453X (▴) and rPC-452X (●). †, further deletions of the PC carboxyl-terminus resulted in undetectable secreted PC protein levels.

We subsequently assessed the impact of the amino acid change on the FIX coagulant properties. To this purpose, we exploited functional aPTT-based assays by supplementing FIX-depleted plasma with the rFIX-containing medium. Whereas the coagulation time upon addition of 25 ng/ml of rFIX-wt was 88.0 ± 0.8 s, that of a similar amount of rFIX-450C was prolonged (105.4 ± 3.3 s), thus leading to estimate a specific activity of 0.14 ± 0.006 (Fig. 3A).

Taken together these data demonstrated that introduction of cysteine at position 450 not only severely impairs biosynthesis but also affects coagulant function, thus causing a severe Hemophilia B form.

### 3.2 The phenyl group of tyrosine 450 (c234) has a key role for secretion and function

Analysis of the primary sequences of serine proteases revealed that the phenyl group at position c234 is highly conserved [10]. Taking into account the highly homologous coagulation serine proteases, tyrosine is present in FVII, FIX and PC but not in FX, which displays phenylalanine (Fig. 1). Moreover, previous studies on the rFIX-450H and rFIX-450P variants, whose secretion levels were reduced to 7% and undetectable [11], respectively, further support the detrimental impact of amino acid changes at this position. On the other hand, inspection of the crystallographic structure of activated FIX (ID 1RFN) suggests that tyrosine 450 is partially exposed on the serine protease domain. The cysteine sulfhydryl group introduced by the p.Y450C mutation could lead to illegitimate disulfide bridges, and/or induce FIX dimerization in the endoplasmic reticulum, thus contributing to the biosynthetic impairment. To address this issue, we performed Western blotting analysis in non-reducing conditions of cell lysates, which did not reveal FIX isoforms compatible with the presence of dimers (Fig. 2A, inset). Moreover, we did not observe appreciable quantitative differences between the rFIX-wt and rFIX-450C. This observation is consistent with the intracellular rFIX-450C levels measured by ELISA (112.8 ± 2.6% of rFIX-wt), which excludes its intracellular accumulation and is compatible with a quick degradation of the mutant protein by the quality control system.

In an attempt to dissect the biochemical bases of our experimental observations, we studied the effects on FIX biology of the introduction of phenylalanine and serine (Fig. 1), maintaining the phenyl ring or the oxydryl groups of tyrosine, respectively. Similarly to the natural rFIX-450C variant, the secretion of the rFIX-450S was markedly reduced (5.5 ± 0.2% of rFIX-wt) and delayed (Fig. 2). At variance, the rFIX-450F was secreted in medium at appreciable levels (25.3 ± 2.3%).

Functional assays in plasma systems revealed remarkable differences among mutants. In particular, the specific activity of the rFIX-450S (0.15 ± 0.05) was strongly reduced and comparable to that of the natural rFIX-450C variant, while the rFIX-450F variant displayed a virtually normal specific activity (0.78 ± 0.19) (Fig. 3A).

These findings indicate that the removal of the phenyl ring, rather than the introduction of the sulfhydryl group, has the major detrimental effect and is responsible for both impaired secretion and dysfunctional FIX molecules. The importance of the phenyl rings in the FIX carboxyl-terminus is further highlighted by the observation that substitutions involving the other two tyrosine residues of this region (p.Y441C and p.Y444S) are associated to Hemophilia B [20], [21]. However, the absence of information on the circulating FIX protein levels in these patients does not permit a proper comparison with the mutation under study.

Interestingly, for the mutations at position 450 not preserving the phenyl ring the two detrimental effects on secretion and function were not disentangled. Their direct relation implies an additive behavior, which produces a steep gradient of function impairment (Fig. 3A).

### 3.3 Differential impact of alterations in the carboxyl-terminal region of homologous coagulation serine proteases

The present findings on FIX differ substantially from our observations in FVII [14], and from those obtained in PC [13]. Indeed, substitutions of R462 (c253) or short deletions at the FVII carboxyl-terminus resulted in reduced secreted levels of proteins with normal specific activity (Fig. 3B). The R462X paradoxically displayed gain-of-function features that produced an asymptomatic FVII deficiency form. Noticeably, these observations indicate that additive or compensatory pleiotropic effects elicited by mutations in the carboxyl-terminus of FIX and FVII, which similarly reduce the circulating protein amount, give rise to life-threatening or mild phenotypes. On the other hand, a deletion scanning of the carboxyl-terminal region of PC revealed that alterations of this protein region have a major impact on secretion, as indicated by the normal specific activity of the secreted truncated PC variants (Fig. 3B).

Taken together these information suggest that the carboxyl-terminal region of these highly homologous proteins [22] does not represent a sole secretion determinant, but has a still undefined role for FIX and FVII function.

## 4 Conclusions

The study of model mutations in the carboxyl-terminus of FIX is particularly informative to elucidate the interplay between pathogenic molecular mechanisms impairing protein biosynthesis and function. We provide evidence for a dual role of the FIX carboxyl-terminus for both secretion and coagulant activity and for additive effects of the p.Y450C mutation, which explain a particularly severe Hemophilia B form. Comparison of findings in the highly homologous coagulation serine protease family members suggests that variations in this protein region might have contributed to the evolution of this protein family sub-group.

## Financial support

The study was supported by University of Ferrara, Telethon – Italy (GGP09183), Ministero dell’Universita‘ e della Ricerca (MIUR)-Progetti di Ricerca di Interesse Nazionale (PRIN) and AIFA (AIFA 2008 – Bando per le malattie rare – Progetto RF-null-2008-1235892).
